# Exploring uncertainty and use of real-world data in the National Institute for Health and Care Excellence single technology appraisals of targeted cancer therapy

**DOI:** 10.1186/s12885-022-10350-8

**Published:** 2022-12-05

**Authors:** Jiyeon Kang, John Cairns

**Affiliations:** 1 Department of Health Service Research and Policy, Faculty of Public Health and Policy, London School of Hygiene and Tropical Medicine, 15-17 Tavistock place, London, WC1H 9SH UK; 2 Centre for Cancer Biomarkers (CCBIO), University of Bergen, Bergen, Norway

**Keywords:** NICE, HTA, Targeted cancer therapy, Uncertainty, RWD

## Abstract

**Objectives:**

Dealing with uncertainty is one of the critical topics in health technology assessment. The greater decision uncertainty in appraisals, the less clear the clinical- and cost-effectiveness of the health technology. Although the development of targeted cancer therapies (TCTs) has improved patient health care, additional complexity has been introduced in drug appraisals due to targeting more specific populations. Real-world data (RWD) are expected to provide helpful information to fill the evidence gaps in appraisals. This study compared appraisals of TCTs with those of non-targeted cancer therapies (non-TCTs) regarding sources of uncertainty and reviewed how RWD have been used to supplement the information in these appraisals.

**Methods:**

This study reviews single technology appraisals (STAs) of oncology medicines performed by the National Institute for Health and Care Excellence (NICE) over 11 years up to December 2021. Three key sources of uncertainty were identified for comparison (generalisability of clinical trials, availability of direct treatment comparison, maturity of survival data in clinical trials). To measure the intensity of use of RWD in appraisals, three components were identified (overall survival, volume of treatment, and choice of comparators).

**Results:**

TCTs received more recommendations for provision through the Cancer Drugs Fund (27.7, 23.6% for non-TCT), whereas similar proportions were recommended for routine commissioning. With respect to sources of uncertainty, the external validity of clinical trials was greater in TCT appraisals (*p* = 0.026), whereas mature survival data were available in fewer TCT appraisals (*p* = 0.027). Both groups showed similar patterns of use of RWD. There was no clear evidence that RWD have been used more intensively in appraisals of TCT.

**Conclusions:**

Some differences in uncertainty were found between TCT and non-TCT appraisals. The appraisal of TCT is generally challenging, but these challenges are neither new nor distinctive. The same sources of uncertainty were often found in the non-TCT appraisals. The uncertainty when appraising TCT stems from insufficient data rather than the characteristics of the drugs. Although RWD might be expected to play a more active role in appraisals of TCT, the use of RWD has generally been limited.

**Supplementary Information:**

The online version contains supplementary material available at 10.1186/s12885-022-10350-8.

## Introduction

In England, the National Institute for Health and Care Excellence (NICE) has a role in assessing health technology, such as drugs and medical devices, in informing the best value of using the National Health Service (NHS) resources. Cost-utility analysis is the primary method to assess value for money in appraisals of cancer treatments. Uncertainty is unavoidable when appraising the clinical- and cost-effectiveness of new drugs. Uncertainty refers to the fact that we do not know the expected costs and effects of an intervention in a particular population of patients with absolute precision [1] — the more uncertainty there is in the clinical and cost-effectiveness evidence base for a health technology, the less clear is the appropriate decision. Limited clinical evidence, such as non-comparative studies, studies with small numbers of patients and studies with limited follow-up, could be sources of increased uncertainty in health technology assessment (HTA) decision-making [2]. Although data are not sufficient, a decision must still be made. Charlton highlighted that NICE has made decisions based on weaker evidence than previously, which can diminish fairness [3]. Hence, understanding and dealing with uncertainty has become more critical than ever in HTA, given the increasing use of uncertain evidence.

Targeted cancer therapy (TCT) refers to treatments that act on specific molecules associated with cancer growth, progression and spread guided by biomarker results [4]. Lung cancer is one of the cancers for which TCTs are actively developed. Several altered driver oncogenes characterise non-small cell lung cancer, including KRAS, EGFR, ROS1, ALK, and MET exon 14 alterations [5]. These biomarkers are actively used to develop the targeted therapy. Most of the latest lung cancer treatments are targeted therapies [6]. Over the last decades, TCT has aroused interest because of the prospect of achieving better health outcomes [7]. TCT selects a treatment population based on the expression of biomarkers. Such population targeting can introduce appraisal challenges, for instance recruiting an adequate sample size in clinical trials or choosing relevant comparators based on patient stratification [8, 9]. In some trials, subgroups are used to show the clinical effectiveness with a suitable biomarker expression. However, subgroups are likely to be too small to demonstrate statistical significance. These challenges potentially make clinical trials less generalisable to NHS clinical practice. Ultimately, they are likely to be potential sources of uncertainty in appraisals of TCT [10].

Real-world data (RWD) are suggested as a means of overcoming evidence gaps and helping appraisal of innovative drugs in light of the challenges of obtaining the required information from randomised controlled trials (RCTs) [11, 12]. For example, electronic health records (EHR), a form of RWD, are a potential source of mature survival data which can reduce uncertainty regarding long-term outcomes [13]. Also, the use of RWD has been highlighted as a means of constructing external control arms and supporting indirect treatment comparison in decision-making when the treatment effectiveness of comparators is not available from clinical trials [14, 15]. Furthermore, RWD could provide clinical and environmental information at the patient level, reflecting routine practice [16].

The uncertainty in appraisals is one of the significant concerns in HTA decision-making. RWD has received attention as a means of reducing uncertainty. However, there are caveats with using RWD due to confounders, biases and data quality [17]. Also, it is unclear whether RWD can provide the appropriate information in an HTA decision-making context. The Cancer Drugs Fund (CDF) in England offers patients access to drugs while collecting additional information to reduce uncertainty using managed access agreements [18]. A recent paper has highlighted RWD’s limited role in reducing uncertainty in CDF review appraisals [19]. Despite awareness of uncertainty in TCT appraisals and the potential for using RWD, it is unknown to what extent the uncertainties differ between appraisals of TCT and non-TCT and whether RWD are more widely used in economic evaluations of TCT. This study compares appraisals of TCT and non-TCT regarding sources of uncertainty and reviews the use of RWD in these appraisals.

## Method

This study compared single technology appraisals (STAs) of TCT and non-TCT in terms of appraisal recommendations, the size of clinical trials, types of uncertainties and use of RWD. Chi-square tests were used to show whether any differences between TCT and non-TCT were statistically significant. This analysis includes NICE STAs of oncology medicines for which guidance was issued between January 2011 and December 2021 (*n* = 229). NICE technology appraisal guidance is publicly available (https://www.nice.org.uk/guidance). The appraisals were manually screened to identify the relevant appraisals. This study uses data extracted following a protocol developed to record information about the use of RWD in NICE appraisals of oncology medicines [20]. This protocol was designed to extract data used in the economic evaluation, such as general information about technology appraisals, primary clinical evidence characteristics, and the use of RWD. All necessary data for the analysis are available from this dataset.

This research required a definition of TCT. One broadly accepted definition is a cancer treatment that targets specific genes and proteins involved in the growth and survival of cancer cells. However, the definition of TCT has changed over time [21], and TCT, precision medicine and personalised medicine are used interchangeably. Moreover, a biological definition of targeted therapy is less relevant to capture the issues when appraising TCT, as targeting biological molecules does not directly cause the problem. The issues often arise from specifying the population using biomarkers. Hence, in this paper, TCT is defined as an anti-cancer therapy where the indication approved by medical regulators distinguishes patients using biomarkers. In contrast, non-TCT is a cancer treatment not defined as TCT. This implies that some drugs can be categorised differently depending on the indication.

Any analysis of NICE recommendations needs to recognise that a new option became available in 2016 with the advent of a revised CDF. As the available options differ, this study reviewed the NICE appraisal recommendations separately before and after the 2016 CDF. The revised 2016 CDF was introduced in April 2016. The first STA of a cancer medicine after the 2016 CDF was introduced was the appraisal of azacitidine for treating acute myeloid leukaemia with more than 30% bone marrow blasts (TA399). Any STAs issued after TA399 were regarded as ‘after 2016 CDF’.

The size of clinical trials was also reviewed in this study. The number of patients included in the trials was summarised in a histogram to look at the distribution of the trial size. Kernel Density estimation was used to approximate the histogram with a continuous distribution. This estimation compared the similarities and differences between TCT and non-TCT appraisals, focusing on the average number of patients in the trials.

This study focuses on three potential sources of uncertainty in NICE appraisals: the external validity of clinical trials, the availability of direct treatment comparisons, and the maturity of survival data. The sources of uncertainty identified by Morrell et al. [22] were classified into three groups. Appraisal Committees often discuss these sources of uncertainty. The external validity of the clinical study to NHS practice is assessed primarily using the Evidence Review Group’s (ERG) assessment of external validity, which the authors have used to classify studies into three groups (acceptable, moderate, and questionable external validity). Three issues potentially affecting external validity (appropriateness of comparators, subsequent treatments received by trial participants, and patient characteristics) are selected to discuss external validity [23, 24]. When one or more of these issues is identified, the study is coded as of *questionable external validity*. External validity is considered moderate if the ERG raises a few minor concerns. A comment such as “younger and fitter patients” without mentioning performance status is classified as a minor concern. External validity is classified as *acceptable* if there are no specific critiques.

The type of treatment comparison made by manufacturers in their evidence submissions is reviewed to identify the availability of direct treatment comparisons. A sixfold classification of treatment comparisons in NICE appraisals can be made using the information on the availability of head-to-head comparison for all comparators, indirect treatment comparison, anchored/unanchored treatment comparison and population-adjusted treatment comparison. The possible combinations of treatment comparison are presented in Additional file 1: appendix 1.

Lastly, the maturity of survival data is highlighted as a source of uncertainty. This study uses three categories (extremely immature, immature, mature) based on the percentage of death events in the primary clinical studies. 20 and 50% were used in this study to classify appraisals, adapting the findings from Tai et al. [25]. If the proportion of death events is less than 20%, the maturity of survival data is recorded as extremely immature. When the proportion of death events is between 20 and 50%, the survival data are immature, and greater than 50%, the survival data are considered mature. The published clinical studies were consulted if this information was redacted in the appraisal document. If the proportion was not reported in the results of the original research, comments on maturity in the ERG report were checked. If none of this information was available, the survival data were considered extremely immature.

**Fig. 1:**
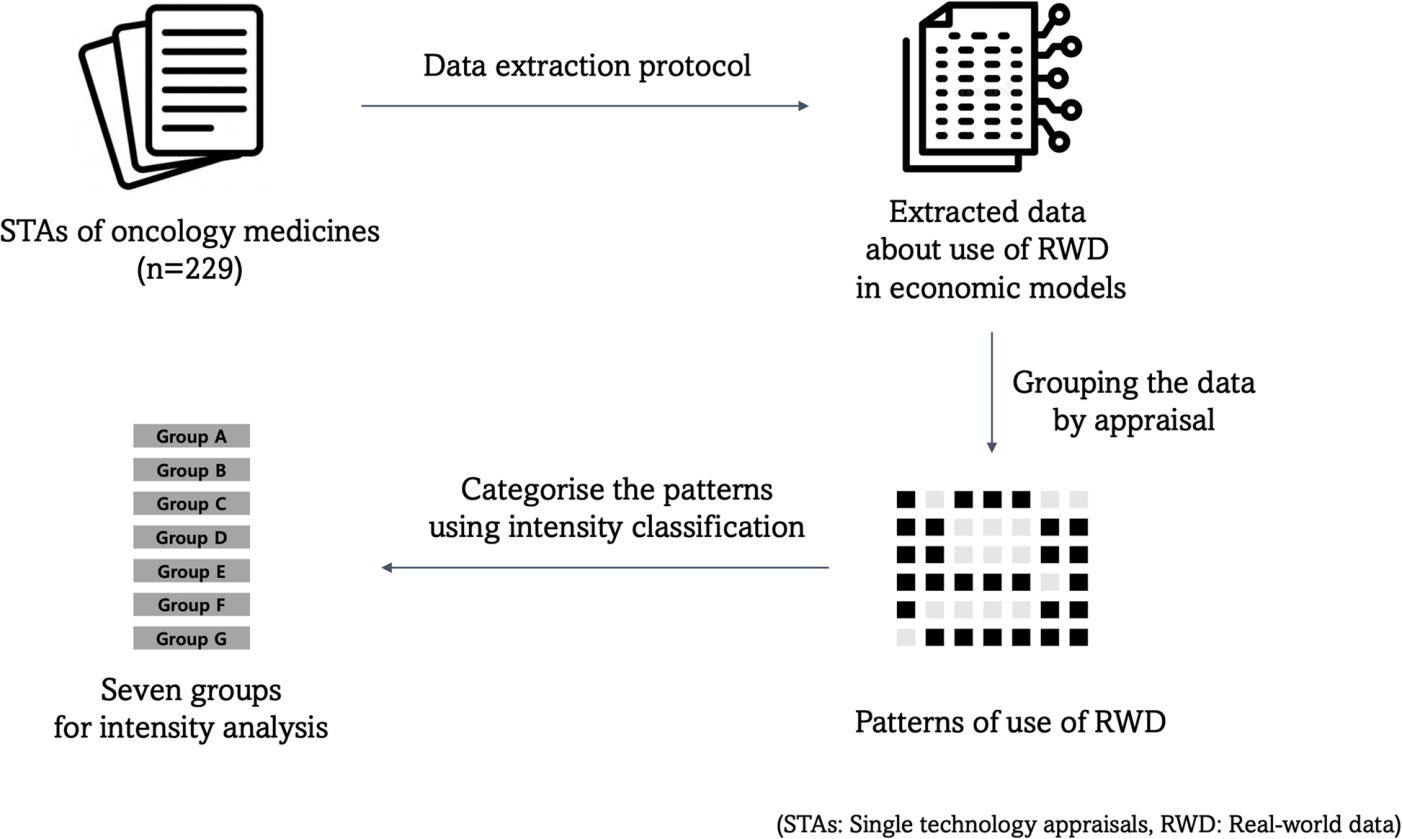
Diagram of data preparation

There are many potential uses of RWD in an appraisal and several ways of reporting the use of RWD. Simple counts of the number of occasions when RWD are used in an appraisal may not be a good guide to how differently one appraisal utilises RWD compared to another. This study used a few different methods, such as pattern review and intensity analysis, to review the use of RWD. Figure 1 summarises how the data were prepared for these analyses.

The patterns of use of RWD were reviewed to provide a clearer picture of how RWD have been used. The extraction protocol distinguished 31 economic evaluation components where RWD might be used, giving rise to many different patterns. The patterns were reviewed by distinguishing between the parametric and non-parametric use of RWD. Parametric use involves basing the numerical value of specific variables in the economic model on RWD. For example, the use of data to provide values for overall survival (OS) or resource use in the economic model is categorised as parametric use. Non-parametric refers to using RWD to develop the model structure and support or validate assumptions in the model. Using RWD to select comparators or validate the survival distribution choice are examples of non-parametric use. This separation provides a more comprehensive review of how RWD have been used in appraisals. All components where RWD could be used are presented in Additional file 1: appendix 2.

**Fig. 2:**
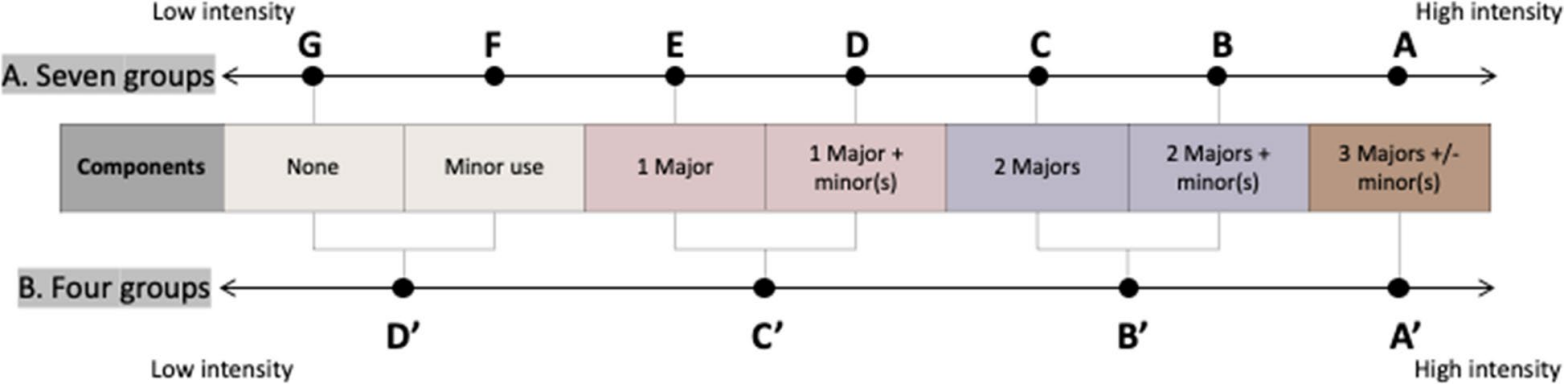
Classifications distinguishing major and minor use of RWD

The intensity of use of RWD in different appraisals was investigated by classifying different patterns in terms of the extent to which RWD are drawn upon in different economic evaluation components. Three components (OS of intervention/comparator, volume of treatment of intervention/comparators, choice of comparators) are identified as major uses of RWD, which are likely to have a high impact on the outcome of the economic evaluation, the incremental cost-effectiveness ratio (ICER). The remaining components are regarded as minor uses of RWD. The identified patterns were categorised into seven groups by distinguishing major and minor uses of RWD (Fig. 2). Two classifications are suggested. One counts the number of major and minor components; another is a simplified classification that only counts the number of major components. The group with all three major components is the highest intensity use of RWD.

## Results

1. Single Technology Appraisals of TCT and non-TCT

**Fig. 3:**
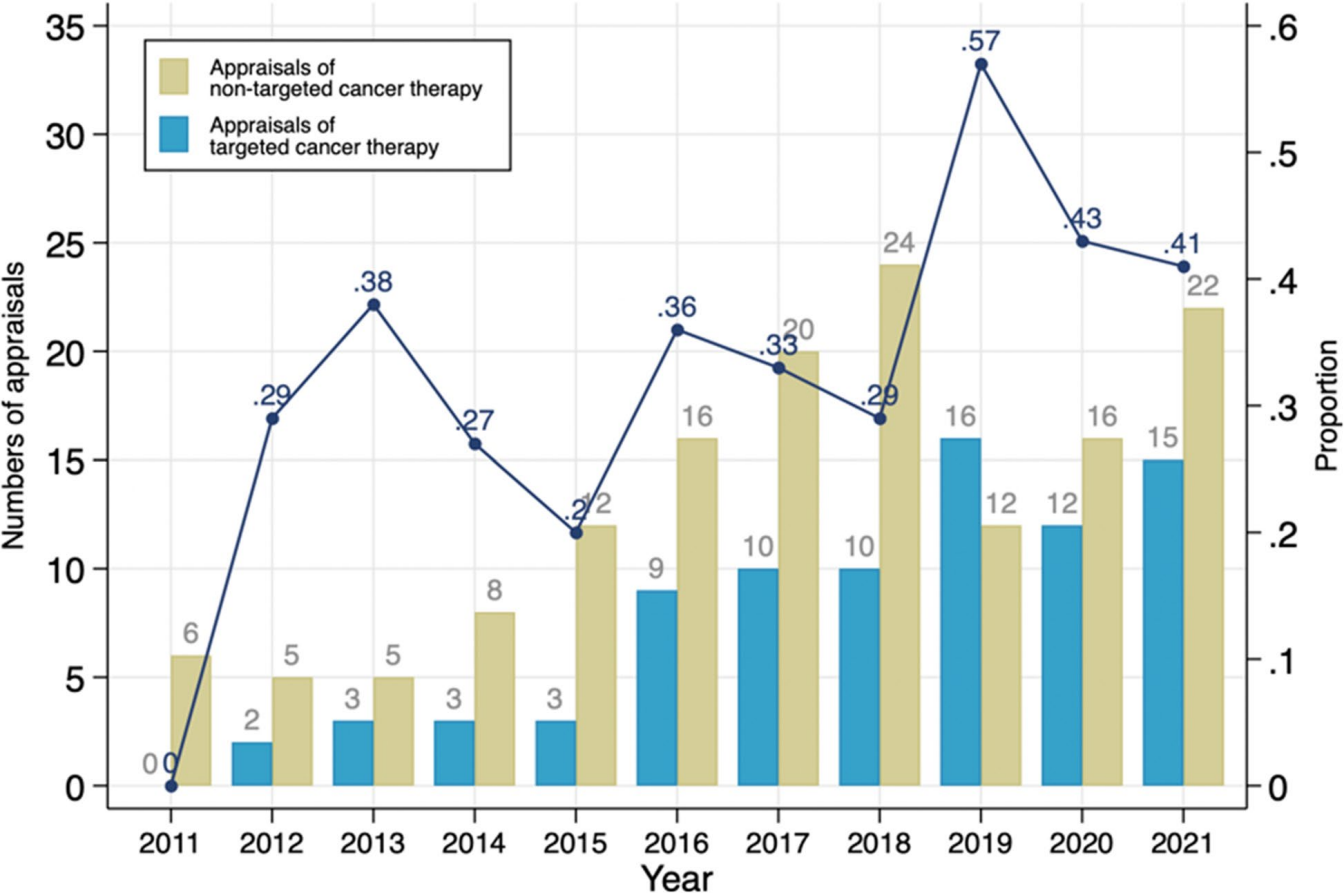
Appraisals of oncology drugs 2011–2021

Figure 3 shows published STAs of TCT and non-TCT over time. All identified STAs were included in this analysis (*n* = 229). The number of STAs of oncologic medicines has generally increased over time except for 2019 and 2020. Of included STAs, 36% were TCT appraisals. Although there were fluctuations, the TCT proportion has increased over time. The highest proportion of TCT appraisals was in 2019–57% of oncology appraisals. Note there were no TCT appraisals published in 2011.

**Fig. 4:**
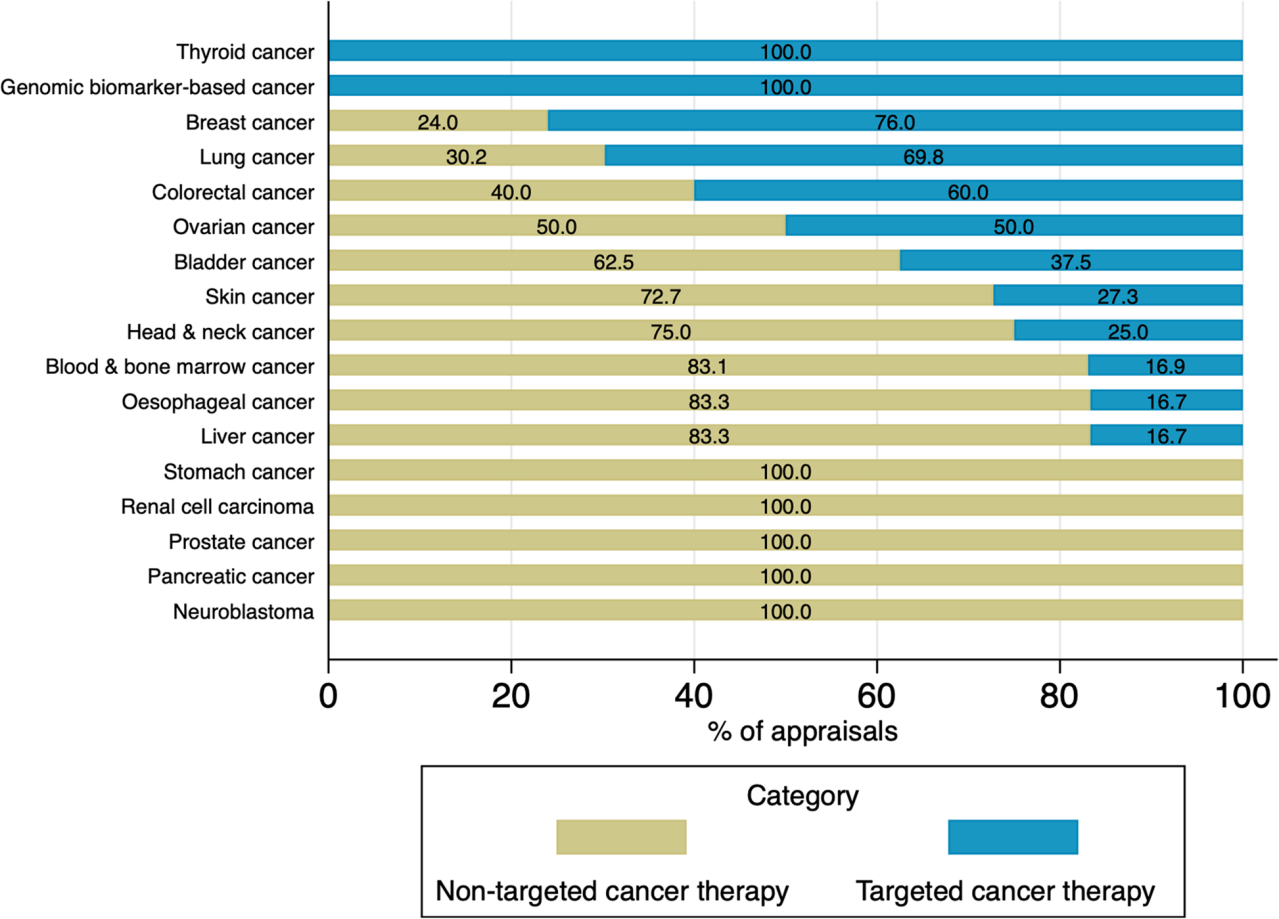
Targeted and non-targeted cancer appraisals 2011–21 by cancer

Figure 4 shows TCT and non-TCT appraisals by cancer type. Cancer areas where TCTs have been actively introduced are breast cancer (76% of breast cancer appraisals) and lung cancer (70% of lung cancer appraisals). In genomic biomarker-based cancer treatments known as histology-independent therapies, TCTs show the highest proportion because of the nature of the treatment. As a new generation of treatment, the genomic biomarker-based cancer treatment is histology-independent, which treats cancers based on a biomarker, not by the location of cancer. The two drugs, entrectinib and larotrectinib in this category, are currently recommended within the CDF.

The TCT and non-TCT appraisal recommendations are reported in Table 1. Overall, appraisals of TCT have a higher proportion of positive recommendations for routine commissioning, although the difference is not statistically significant. There has been no significant difference in recommendations to provide through the CDF between the two groups following the introduction of the 2016 CDF.

**Table 1:** Appraisal recommendations

	TCT	Non-TCT	χ^2^ (p)
Overall			
Not recommended	7 (8.43%)	29 (19.86%)	6.7409 (0.150)
Recommended (routine commissioning)	40 (47.95%)	61 (41.78%)	
Optimised^a^	16 (19.28%)	30 (20.55%)	
CDF	14 (16.87%)	21 (14.38%)	
CDF, Optimised	6 (7.23%)	5 (3.42%)	
Total	83 (100%)	146 (100%)	
Before introducing the 2016 CDF			
Not recommended	3 (23.08%)	15 (38.46%)	1.2966 (0.523)
Recommended	9 (69.23%)	20 (51.28%)	
Optimised	1 (7.69%)	4 (10.26%)	
Total	13 (100%)	39 (100%)	
After introducing the 2016 CDF			
Not recommended	4 (5.71%)	14 (13.08%)	3.8190 (0.431)
Recommended (routine commissioning)	31(45.83%)	41 (38.32%)	
Optimised	15 (21.43%)	26 (24.30%)	
CDF	14 (20.00%)	21 (19.63%)	
CDF, Optimised	6 (8.57%)	5 (4.67%)	
Total	70 (100%)	107 (100%)	

^a^“Optimised” is a recommendation for a smaller group of patients than originally stated by the marketing authorisation

**Fig. 5:**
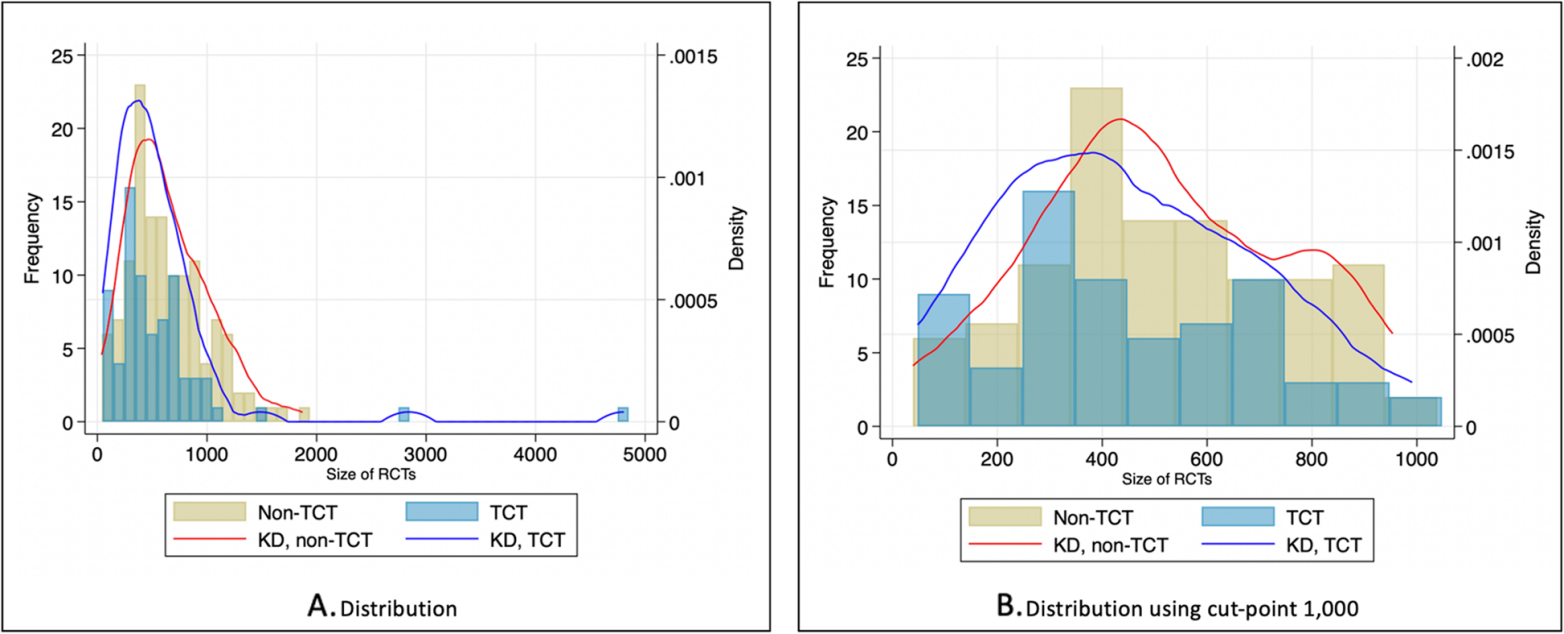
Distribution of trials by size

The number of patients in the clinical trials upon which treatment effectiveness in the economic models was based was reviewed to compare the sizes of the overall trials between TCT and non-TCT. Most clinical studies had fewer than 1000 patients. Right skews were found (Fig. 5A). These right-skewed distributions show that most values for both TCT and non-TCT are clustered around the left tail of the distribution. This distribution implies that most trials (of both TCT and non-TCT) are relatively small. To compare the distributions more clearly, the distributions have been trimmed at 1000 in Fig. 5B. Appraisals of TCT had their peak density around 300–400, whereas appraisals of non-TCT peaked at around 400–500.

2. Sources of uncertainty in NICE appraisals

Potential sources of uncertainty are summarised in Table 2. While there is no statistical difference in the availability of direct treatment comparisons, the external validity of the clinical studies and the maturity of the survival data differ significantly.

**Table 2:** Sources of uncertainty in STAs

	TCT	Non-TCT	χ^2^ (p)
The external validity of clinical studies			
Acceptable external validity	36 (43.37%)	39 (26.71%)	7.2714 (0.026)
Moderate external validity	37 (44.58%)	90 (61.64%)	
Questionable external validity	10 (12.05%)	17 (11.64%)	
Total	83 (100%)	146 (100%)	
Availability of direct treatment comparison			
Not available	28 (33.73%)	43 (29.45%)	1.1922 (0.551)
Some available	28 (33.73%)	45 (30.82%)	
All available	27 (32.53%)	58 (39.73%)	
Total	83 (100%)	146 (100%)	
Maturity of survival data			
Extremely immature	29 (34.94%)	56 (38.36%)	7.2550 (0.027)
Immature	35 (42.17%)	38 (26.03%)	
Mature	19 (22.89%)	52 (35.62%)	
Total	83 (100%)	146 (100%)

1. The external validity of the clinical study

The uncertainties concerning external validity raised in the appraisals were reviewed. These factors (appropriateness of comparators, subsequent treatment received by trial participants, and patient characteristics) are usually addressed in the ERG reports when assessing the generalisability of trial outcomes to NHS practice. Twenty-seven appraisals were identified, where the ERG highlighted the high level of uncertainty with respect to the external validity of the clinical evidence. Ten of these appraisals were TCT. Problems were identified with respect to the study population (70%), the comparators (20%) and subsequent treatment received by trial participants (10%). In appraisals of non-TCT, the external validity of evidence was heavily questioned in seventeen appraisals. The main reason was the study population (53%), followed by the issue of subsequent treatment received by trial participants (35%). The general problem of trial populations being younger and fitter than routine practice is widely noted by ERGs. However, this was not a major reason for the high level of uncertainty unless subgroups in the trial were very different from those in routine practice. More often, the issues with respect to the study population arose from differences in prior treatment, which might impact survival outcomes. For example, in an appraisal of nivolumab (NICE TA530), the ERG expressed serious concerns regarding the representativeness of the trial population to the UK population. One of the reasons was a mismatch of prior therapies. More than 75% of patients in UK clinical practice received a previous gemcitabine platinum-based therapy, while less than 40% of the trial population did. Another example is an appraisal of durvalumab (NICE TA578). The ERG identified that the population in the clinical trial (PACIFIC) was narrower than in the scope (patients expressing PD-L1 > 1%). Also, they received different types of chemoradiation therapy cycles. UK patients received sequential rather than overlapping treatment, potentially affecting the treatment effect.

2) Types of treatment comparison in manufacturer submissions

**Fig. 6:**
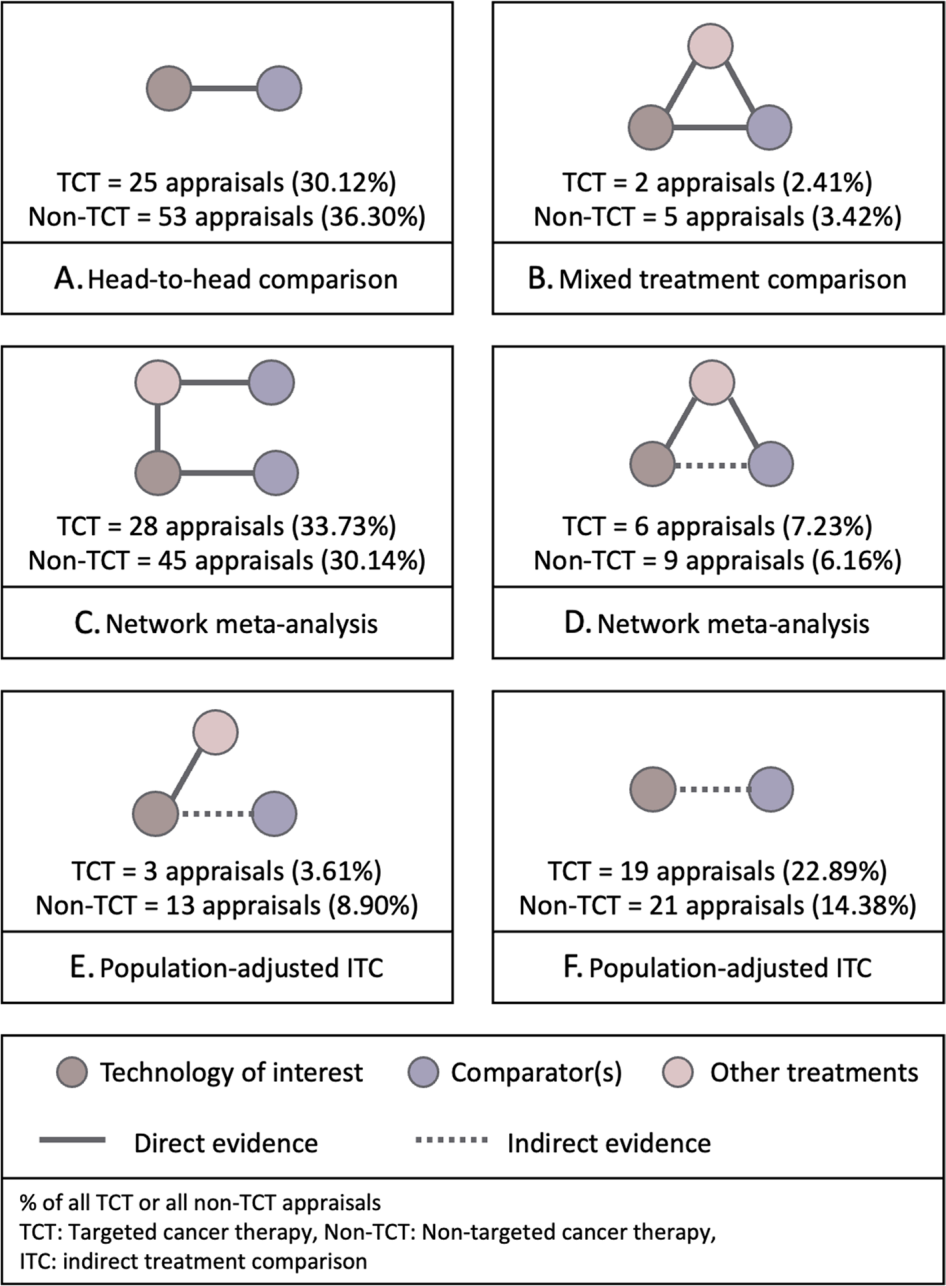
Illustration of treatment comparisons identified in company submissions

The treatment comparisons made were not statistically different between TCT and non-TCT appraisals. The availability of head-to-head RCTs was reviewed to understand the patterns of indirect treatment comparison. The proportion of single-arm trials in TCT appraisals is higher than that of non-TCT. Nineteen TCT appraisals did not use RCTs as primary clinical evidence (23% of TCT appraisals). Several possible ways to compare treatments were found in these appraisals (Fig. 6). In general, TCTs and non-TCTs show similar patterns of treatment comparisons. Thirty-one per cent of all appraisals made indirect treatment comparisons (ITC). Among the appraisals using ITC, 79% made unanchored ITC. TCT appraisals show a higher proportion of unanchored ITCs than non-TCT (23% of TCT appraisals, 14% of non-TCT appraisals).

3) Maturity of survival data in clinical trials

**Fig. 7:**
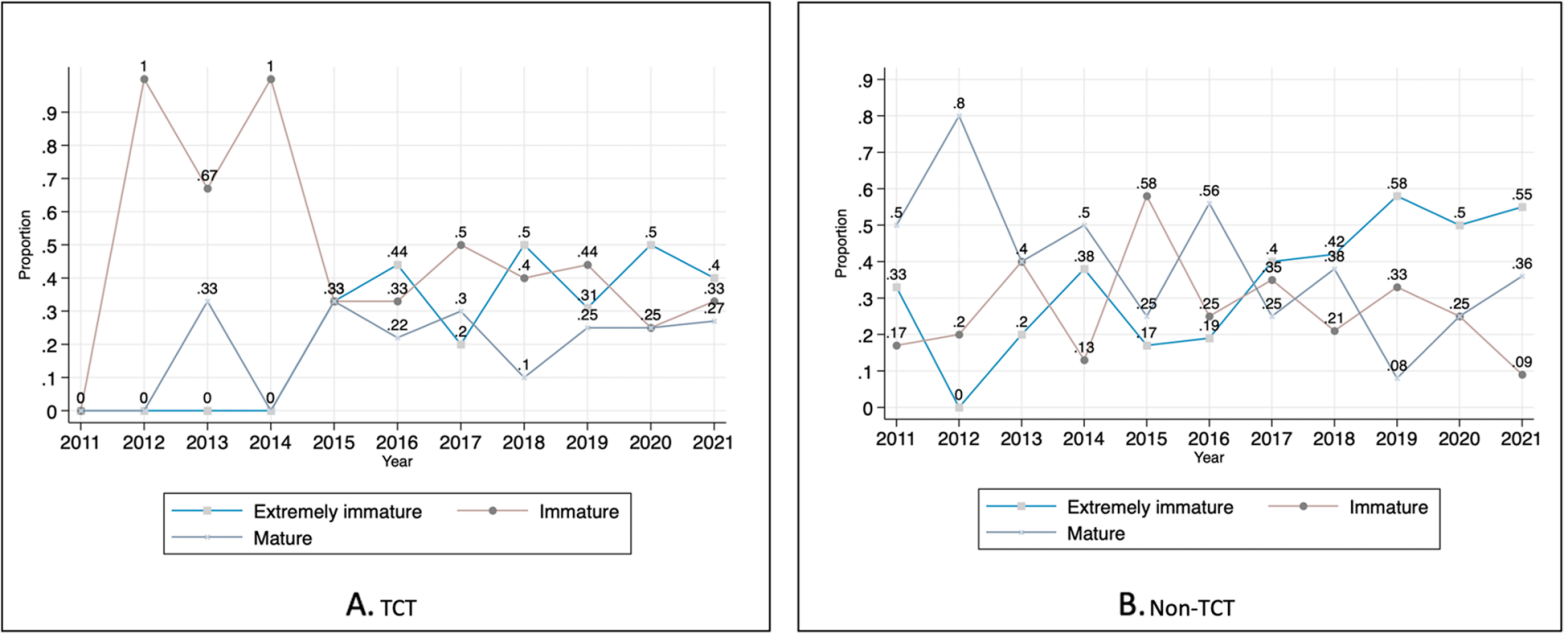
Maturity of survival data in STAs of TCT and non-TCT

The maturity of survival data showed a statistical difference between TCT and non-TCT appraisals. The proportion using extremely immature survival data was similar between the two groups, whereas immature survival data were used more in TCT appraisals. The changes in the use of extremely immature, immature and mature survival data over time are shown in Fig. 7. Although it is difficult to see the clear patterns in the use of immature survival data, the proportion of the STAs using immature survival data tends to have increased over time in both groups.

3. The use of RWD in the economic models of TCT and non-TCT

1. Pattern review

There is no dominant pattern of use of RWD in these appraisals. Fifteen different patterns of use of RWD can be identified, which appeared in three or more appraisals. These patterns cumulatively account for 51% of all appraisals (Additional file 1: Appendix 3). The pattern, estimating overall survival of intervention and comparators, was the most commonly observed (13 appraisals, 6% of patterns), followed by the pattern estimating end-of-life resource use (12 appraisals, 5% of patterns). In appraisals of TCT, using RWD for estimating end-of-life cost is the most common pattern (8 appraisals, 10% of patterns), whereas estimating OS of intervention and comparators was found in only one TCT appraisal (1%).

When looking at the non-parametric and parametric use of RWD separately, more diverse patterns were found for parametric use than for non-parametric use. Sixty-two per cent of all appraisals involved no non-parametric use of RWD (Additional file 1: Appendix 4). The commonest pattern of non-parametric use of RWD was to validate the choice of survival distribution for the intervention and comparators (TCT: 11 appraisals, 13%; non-TCT: 9 appraisals, 6%). Some patterns found in non-TCTs were not identified in appraisals of TCT. Regarding the parametric use of RWD, 23% of appraisals did not use RWD to inform any parameter in the model (Additional file 1: Appendix 5). In appraisals of TCT, using RWD for estimating end-of-life resource use (16 appraisals, 19%) and for estimating both end-of-life and health state resource use (7 appraisals, 8%) were common patterns. Fifteen non-TCT appraisals (10%) used RWD to estimate OS for the intervention and comparators.

2) Intensity analysis

**Fig. 8:**
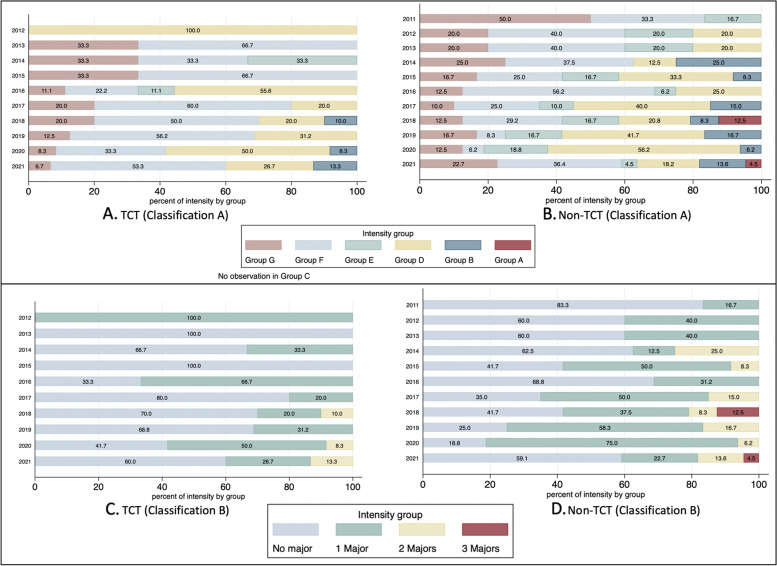
Intensity of use of RWD over time

For analysis of the intensity of use of RWD, all appraisals included in this study were classified into intensity groups using the two classifications in Fig. 2. While classification A shows a statistically significant difference in intensity between appraisals of non-TCT and TCT (χ^2^ = 14.66, *p* = 0.012), classification B does not provide a significant difference (χ^2^ = 6.8035, *p* = 0.078). Over time, the major use of RWD has increased in both groups of appraisals. In 2020, about 60% of TCT and non-TCT appraisals made at least two major uses of RWD. The cases of three major uses of RWD were observed in the non-TCT group in 2018. Such a major use of RWD was not observed in the TCT group. Using classification A (Fig. 8A & B), there does not appear to have been an evident change in the intensity of use of RWD. Whereas, using the simpler classification B, the intensity of use of RWD appears to have increased over time (Fig. 8C & D).

## Discussion

This study compared appraisal recommendations, the size of clinical trials and sources of uncertainty and uses of RWD in STAs of TCT and non-TCT. TCT appraisals have higher rates of positive recommendation, although the difference was not statistically significant. The proportions of positive recommendations might vary in response to differences in the ICERs believed by the appraisal committee. However, the confidential nature of many drug prices limits reporting of precise ICERs and, thus, the exploration of differences in ICERs between TCT and non-TCT. Another possible explanation suggested by Cairns is that uniform pricing across indications combined with individual TCTs having fewer indications might explain the different recourse to the CDF [26]. If a drug is already routinely commissioned for one indication, an extension of routine commissioning to other indications would be expected to be at the original price. In contrast, provision through the CDF could be at a different price.

The size of trials was compared between TCT and non-TCT appraisals. The cancers where TCTs have been actively developed were lung and breast cancer. Both cancers are common cancers [27]. Also, some of the biomarkers found in these cancers are relatively common biomarkers. This implies that the “targeted population” is not necessarily small. Depending on the commonness of the disease and the proportion expressing the relevant biomarker, the target population size could be large enough to show statistical significance. An example is the human epidermal growth factor receptor-2 (HER2) as a prognostic and predictive marker for breast cancer. About 20–30% of breast cancer patients show overexpression of HER2. In the appraisal of trastuzumab emtansine for adjuvant treatment of HER2-positive early breast cancer (TA632), the primary clinical evidence, KATHERINE trial, recruited 1486 patients randomised 1:1 to intervention and comparators. Given that the average trial size of non-TCT was 400–500, in TCT appraisals in these cancers, the extent to which the appraisal challenges are rooted in the characteristics of the TCT is diminished.

In contrast, rare cancers and rare biomarkers, which yield a significantly narrower population, could be a source of the risk when appraising drugs based on highly uncertain evidence in the future. The Neurotrophic tyrosine receptor kinase (NTRK) inhibitors (NICE TA630 larotrectinib, NICE TA644 entrectinib) are good examples of likely future challenges. In these appraisals, the main clinical trials were basket trials, which is a novel trial design to evaluate the treatment effectiveness of TCT for one or more targets regardless of the pathology [28]. Also, companion diagnostic tests for this biomarker were absent [29]. In the entrectinib appraisal, the committee noted that “the population eligible for entrectinib is broader than the trial population, so entrectinib’s clinical effectiveness in some groups is unknown” (p.13, Final Appraisal Determination of NICE TA644). Data from too few patients, immature survival data, and the absence of direct comparison were all addressed in the appraisal. Due to the uncertainty, these drugs are currently recommended within the CDF. Additional data, including RWD, are being collected to reduce uncertainty while these drugs are being provided through the CDF. However, to what extent these additionally collected data will help to reduce uncertainty is not clear [19].

To date, the targeting of treatment populations has not introduced significantly different appraisal challenges. However, the next generation of TCT, such as histology-independent therapy, might present more decision-making challenges, including identifying the eligible population and appropriate prices across the different populations in the future [30]. Overall, TCT appraisals have fewer sources of uncertainty in the evidence despite the concerns about the poor quality of evidence. With respect to uncertainty around external validity, the characteristics of TCTs have some impact on these differences in uncertainty between TCT and non-TCT appraisals. The challenges inevitably increase when the population is restricted using specific biomarkers. Targeting specific populations leads to issues such as insufficient statistical power and eligibility depending on biomarker expression levels, increasing uncertainty regarding the external validity of trial outcomes to NHS practice. However, targeting the population is not the only source of uncertainty in TCT appraisals. Uncertainty is likely to increase with other factors, often found in non-TCT appraisals, such as finding the most suitable population for decision-making. In appraisals of TCT, differences in previous treatment options or subsequent treatment often raised questions concerning the representativeness of the trial data for NHS patients and the likely size of the treatment effect in practice. This adds to the uncertainty around the small size of the eligible population in appraisals of TCT but also of non-TCT.

Uncertain clinical outcomes due to immature survival data are commonly encountered in NICE appraisals [22]. The immaturity of survival data introduces substantial uncertainty in the extrapolation of survival [31, 32]. The TCT appraisals used less mature survival data than appraisals of non-TCT. In appraisals of immunotherapy, a large portion of TCT in this research, appraisal committees often questioned the duration of the treatment effect when predicting the long-term effect. One of the novel response patterns reported in immunotherapy is a sustained response in a small number of patients after stopping immunotherapy [33]. In NICE TA692, the duration of the continued treatment effect was described as an area of uncertainty for all immunotherapies [34]. Immature survival data are more likely to increase the importance of this issue as no long-term data are available. A longer follow-up would help reduce uncertainties concerning the duration of response to treatment and OS [35]. However, this issue is not the only issue in TCT appraisals. A large proportion of non-TCT appraisals used immature survival data. It implies that the absence of long-term data introduces a great level of uncertainty in understanding long-term treatment effects and causes a problem in most cancer appraisals. This can be met by efforts to provide better quality evidence in appraisals and by managed access agreements such as the CDF, which can help to understand the long-term effect by following up the trial population.

The limited availability of direct treatment comparisons was identified as a source of uncertainty across appraisals. Regardless of the treatment type, obtaining head-to-head estimates of comparative effectiveness from a single trial becomes more challenging since the treatment options are rapidly expanding. When direct treatment comparison is not available in a trial, network meta-analysis has been used to identify the treatment effect indirectly. However, a network is not always available unless a common comparator links the available trials [36]. The indirect treatment comparison is unanchored when the primary clinical evidence is a single-arm trial or the evidence cannot be linked to other clinical trials. Analytical techniques such as matching adjusted indirect treatment comparison (MAIC) or simulated treatment comparison have been used when making unanchored comparisons. However, these methods do not usually resolve the uncertainty around indirect comparison since it is not possible to adjust fully for all effect modifiers. An example is the appraisal of trastuzumab deruxtecan (NICE TA704). In this appraisal, the main clinical evidence was a single-arm trial (DESTINY-Breast01). Due to the absence of direct comparative evidence, treatment effectiveness was assessed using an unanchored MAIC. The Appraisal Committee was concerned that important factors such as HER2 status and previous anti-HER2 therapy could not be adjusted for and concluded that the MAIC had limitations and the results were uncertain.

This study found that the evidence used in appraisals of new cancer drugs was uncertain across both TCT and non-TCT appraisals. The sources of uncertainty observed in TCT appraisals were not essentially different from those in appraisals of non-TCT. The uncertainties decision-makers face are ones they have faced previously. Given the novelty of targeted therapy, a new approach was required, such as an innovative clinical trial design and strategy for early decision-making to improve operational efficiency [37]. However, it is uncertain whether novel approaches such as enrichment trial design and trials with adaptive design can help the appraisal process more or introduce additional uncertainty [38, 39]. More importantly, current appraisal challenges arise from data insufficiency rather than the inherent characteristics of these drugs [40]. The sources of uncertainty were more frequently found in the appraisals of non-TCT in this study. Regardless of the type of technology, NICE decision-making uses uncertain evidence.

RWD have been identified as supplementing RCT data. As the pattern review showed, RWD were used in diverse ways. However, while many are optimistic about the potential contribution of RWD [41], the use of RWD has contributed little to both TCT and non-TCT appraisals. RWD were generally only used for relatively unimportant aspects of the evaluation. This limited use of RWD could be explained by several concerns around RWD, including potential bias and study design limitations [42, 43]. Due to the limitations, using RWD might not particularly answer the questions about uncertainty. Also, given that fewer sources of uncertainty were found in TCT appraisals, there could be less incentive to use RWD. Further study of the factors associated with increased/decreased use of RWD would broaden understanding in the future.

Although limited use was made of RWD, it is notable that the intensity of use of RWD has increased over time. Among the patterns that appeared in three or more appraisals, five patterns included using RWD for estimating OS. It is a noteworthy result given the strong signal of NICE’s interest in the use of RWD [44]. Although this study cannot provide detailed information on how RWD were used for this purpose, RWD can be used in several ways to estimate OS, such as adjusting disease hazard and extrapolating the survival curve. Recently, NICE published a real-world evidence framework to guide research on comparative treatment effects using RWD. Additional studies on how RWD have been used in estimating OS will help understand the opportunities and challenges of RWD.

This study explored several aspects of appraisals of TCT and non-TCT from an HTA perspective. Given the increased interest in using biomarkers to identify treatment groups, there will likely be growing challenges in appraising TCT. Although the findings of this study could change over time as more TCT are developed, this study is the first to document systematically the differences and similarities in sources of uncertainty and use of RWD between appraisals of TCT and non-TCT by reviewing over two hundred appraisals. However, this study has a few limitations. First, the information about external validity relies on the ERG reports. Although appraisal committees agree with ERG’s assessments in general, committees do not necessarily always agree on all points with ERGs. What committees critically emphasise regarding external validity could be different.

Another limitation is the classifications of uncertainties and intensity of use of RWD. Although all the information used in this study was obtained from appraisal documents, how to categorise this information was based on the data extraction protocol. The maturity of survival data was classified using two values, 20 and 50%. However, these points are not agreed criteria to define data maturity. Committees can make different judgements with respect to maturity. With respect to the intensity classification, this study focuses on a specific assumption that the use of RWD in three major components would be intensive use of such data. However, the criteria to measure intensity are not universally agreed upon. Also, decision-makers might not be concerned about which RWD inform components of the economic model. More likely, they would concentrate on how RWD would help to address the decision problem. How to classify uncertainty and intensity of use could differ across researchers and decision-makers.

Finally, it is noted that there might be a difference in the number of appraisals depending on which criteria were used. In this study, the STAs for treating side effects of cancer drugs were excluded. When appraisals are collected, potentially also affects their number. Some appraisals available in this study might not be available later due to the replacement of appraisals (CDF review, withdrawn etc.) Likewise, previously available appraisals might not be included in this study as the guidance was withdrawn. Despite this potential difference, this study included all STAs of cancer therapy which were available as of December 2021.

## Conclusion

Some differences in uncertainty were found between TCT and non-TCT appraisals. The appraisal of TCT is generally challenging, but these challenges are neither new nor distinctive. The same sources of uncertainty were also often found in the non-TCT appraisals. The uncertainty in appraising TCTs is more likely to stem from insufficient data rather than the inherent characteristics of the drugs. Although RWD might be expected to take a more active role in appraisals of TCT, the use of RWD has generally been very limited.

## Supplementary Information

**Additional file 1.**

## Data Availability

The data analysed during the current study are available in the National Institute for Health and Care Excellence website: [https://www.nice.org.uk/guidance/published?ngt=Technology%20appraisal%20guidance&ndt=Guidance].

## Abbreviations

CDF: Cancer Drugs Fund
HTA: Health technology assessment
NHS: National Health Service
NICE: National Institute of Health and Care Excellence
Non-TCT: Non-targeted cancer therapy
OS: Overall survival
RWD: Real-world data
TCT: Targeted cancer therapy
