# Supplementary material for: Exploring uncertainty and use of real-world data in the National Institute for Health and Care Excellence single technology appraisals of targeted cancer therapy
Source: BMC Cancer. 2022 Dec 5;22:1268. doi: 10.1186/s12885-022-10350-8 (PMC9724266; doi:10.1186/s12885-022-10350-8)
Supplement: Supplementary file 1 — Additional file 1. [file 12885_2022_10350_MOESM1_ESM.docx]

Appendix 1 Possible combination of treatment comparison in STAs of oncological medicine

| **Types of treatment comparison** | **Illustration** | **Direct treatment comparison** | **Indirect treatment comparison** | **Availability of RCT** | **Anchored comparison** | **Population adjusted comparison** |
| --- | --- | --- | --- | --- | --- | --- |
| Head-to-head comparison | 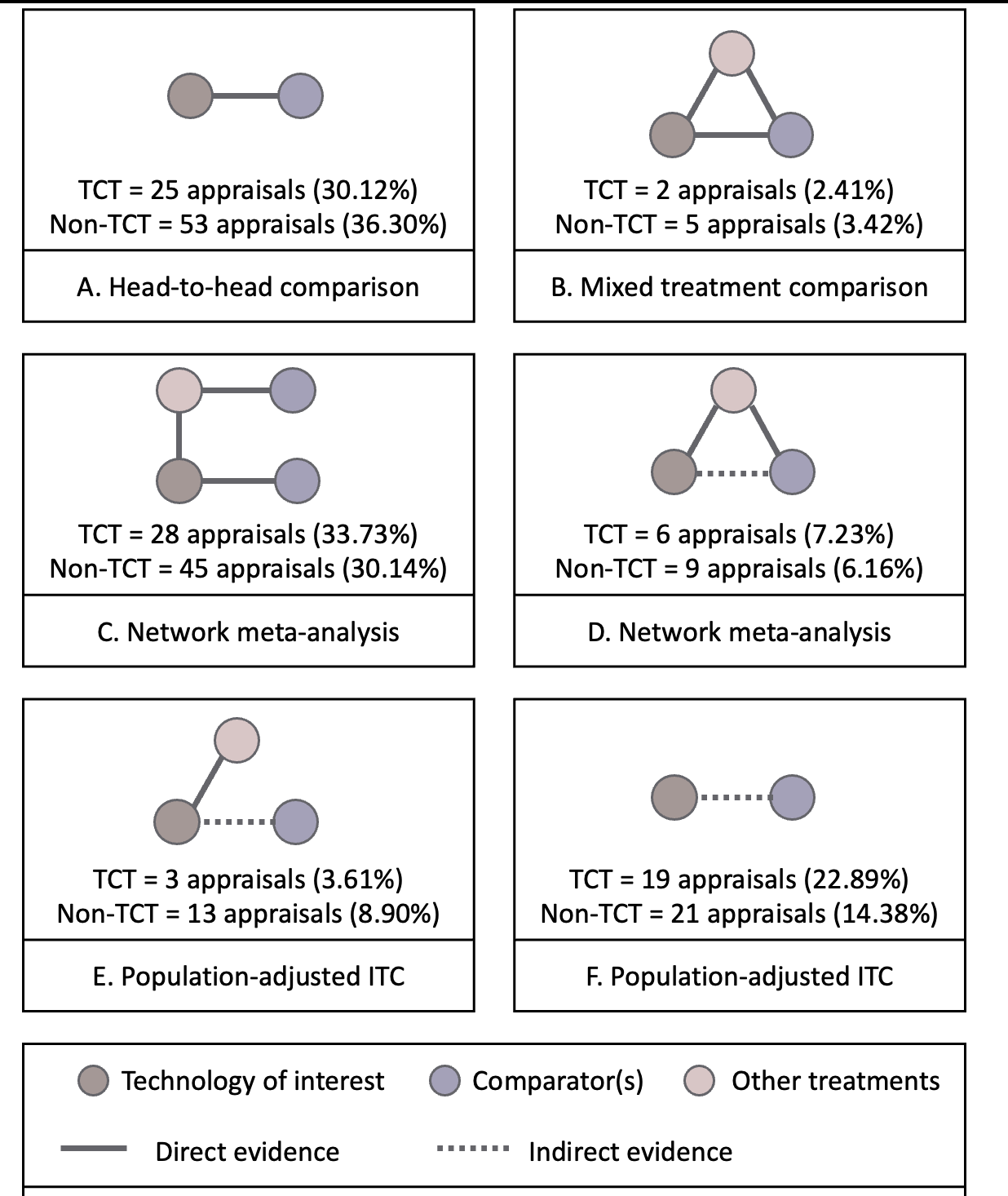 | Yes, all available | Not used | Yes, available | Yes | No |
| Mixed treatment comparison | 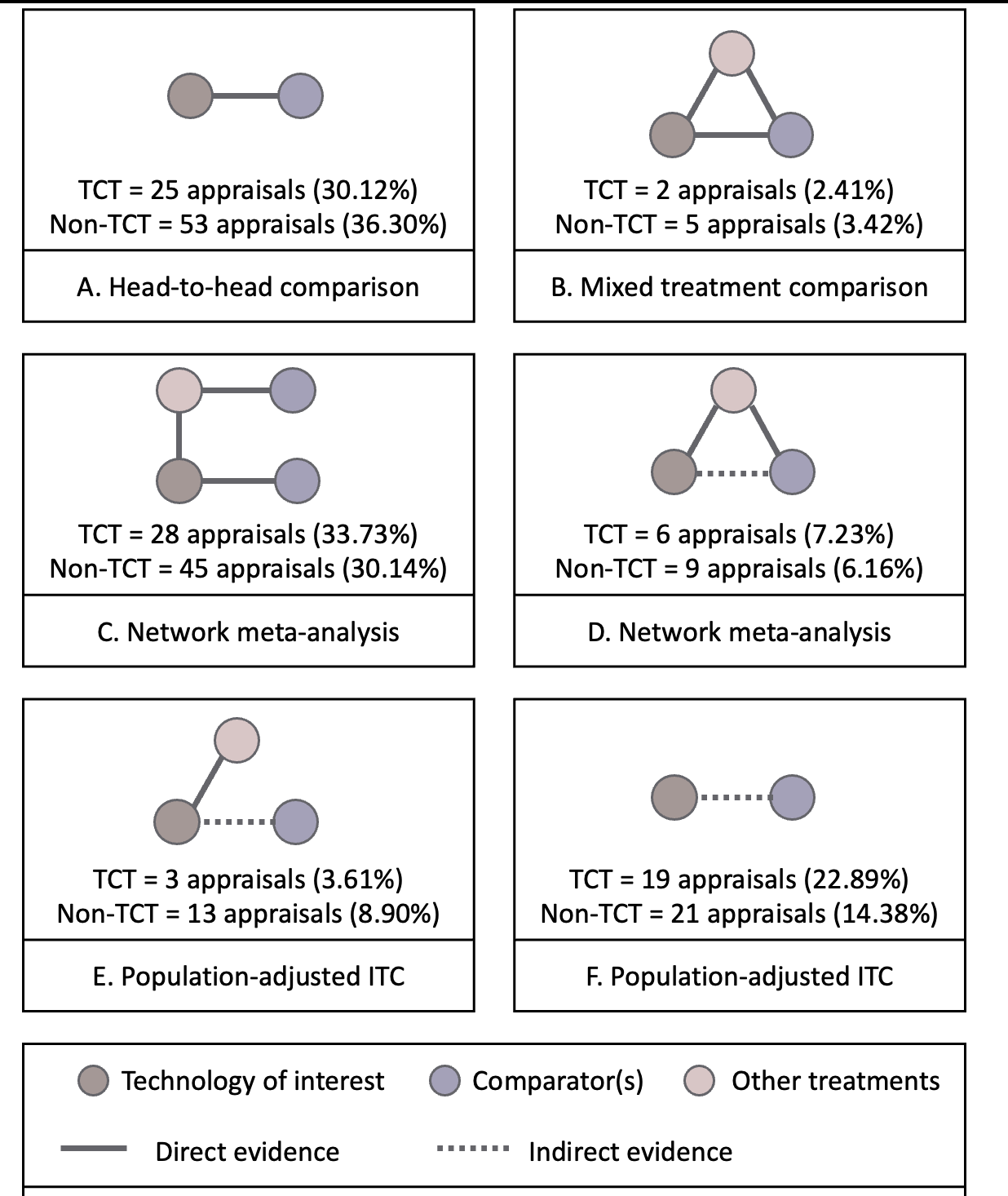 | Yes, all available | Yes, used | Yes, available | Yes | No |
| Population-adjusted indirect treatment comparison | 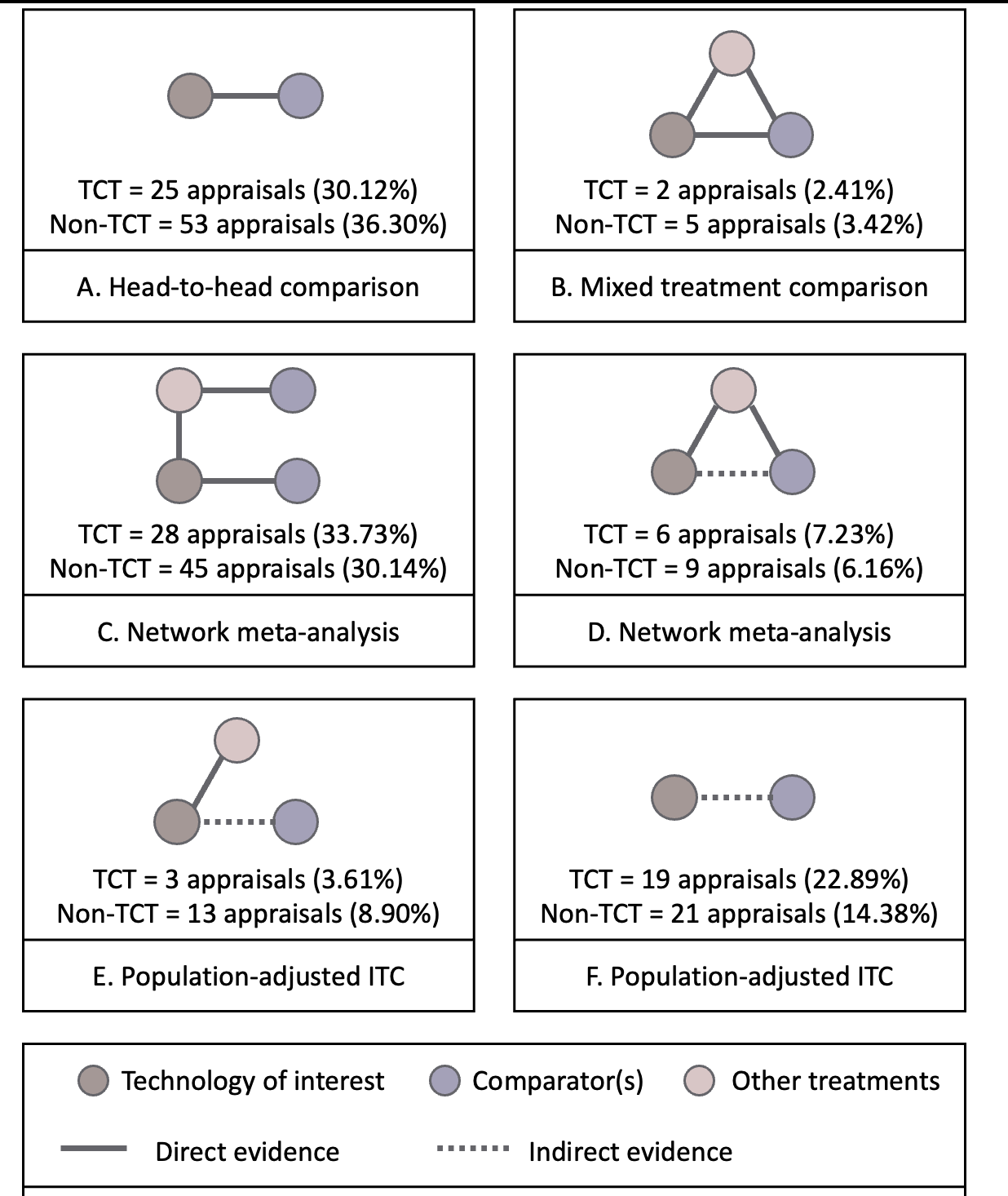 | No, not available | Yes, used | No, not available | No | No (Naïve) |
|  |  |  |  |  |  | MAIC |
|  |  |  |  |  |  | STC |
|  |  |  |  |  |  | Other methods |
|  | 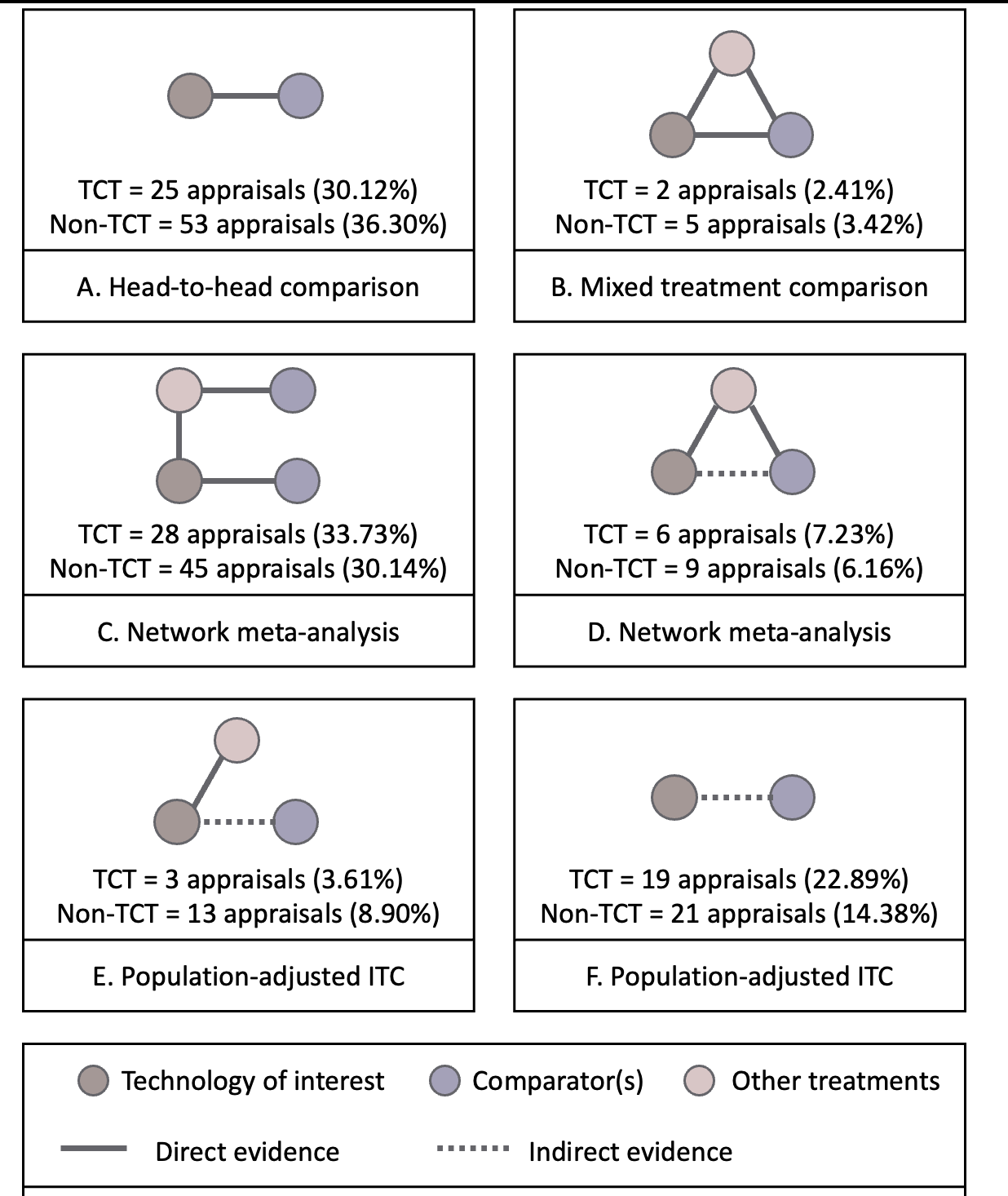 | No, not available | Yes, used | Yes, available | No | No (Naïve) |
|  |  |  |  |  |  | MAIC |
|  |  |  |  |  |  | STC |
|  |  |  |  |  |  | Other methods |
| Network meta-analysis | 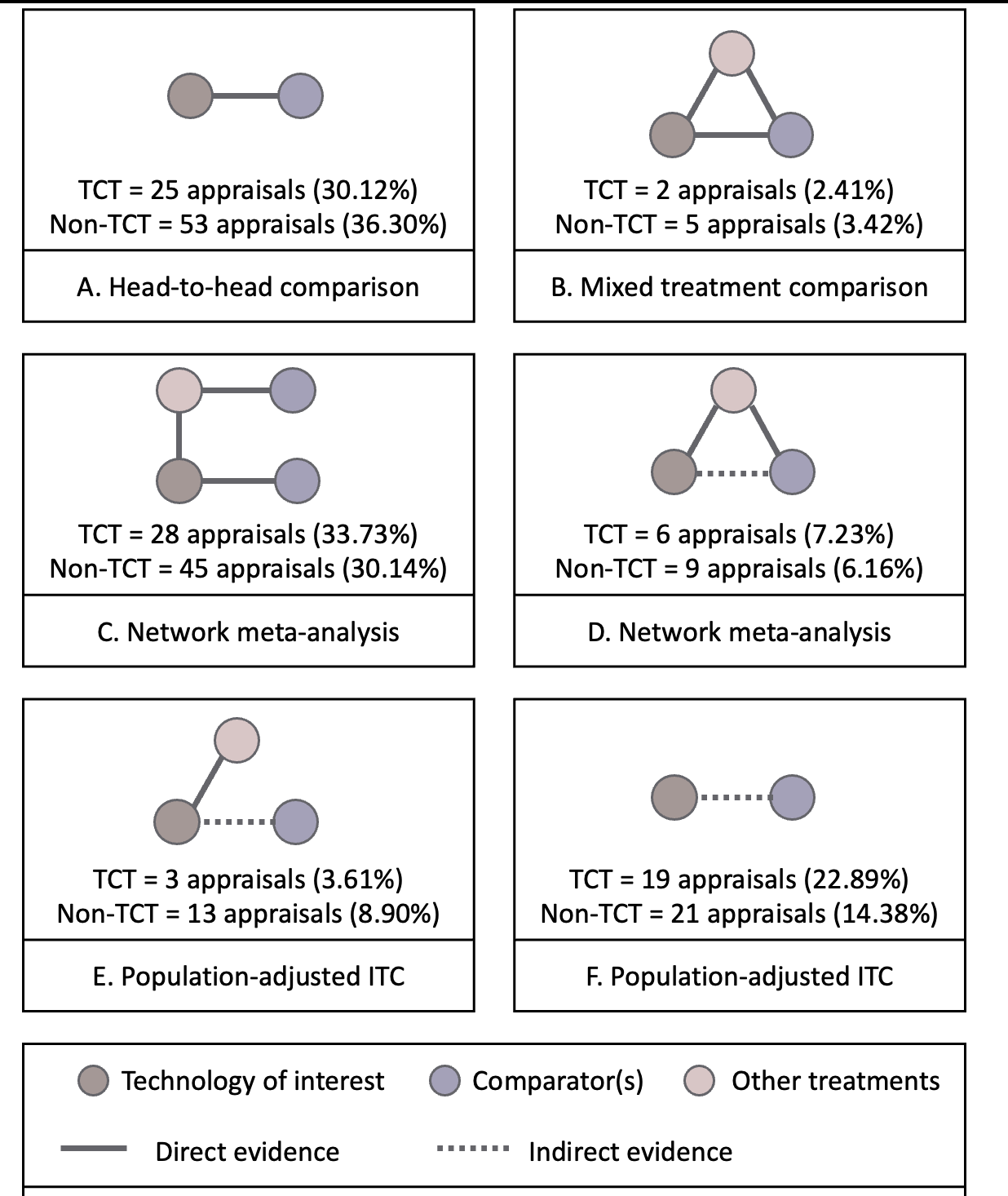 |  |  |  | Yes | No (Naïve) |
|  |  |  |  |  |  | MAIC |
|  |  |  |  |  |  | STC |
|  |  |  |  |  |  | Other methods |
|  | 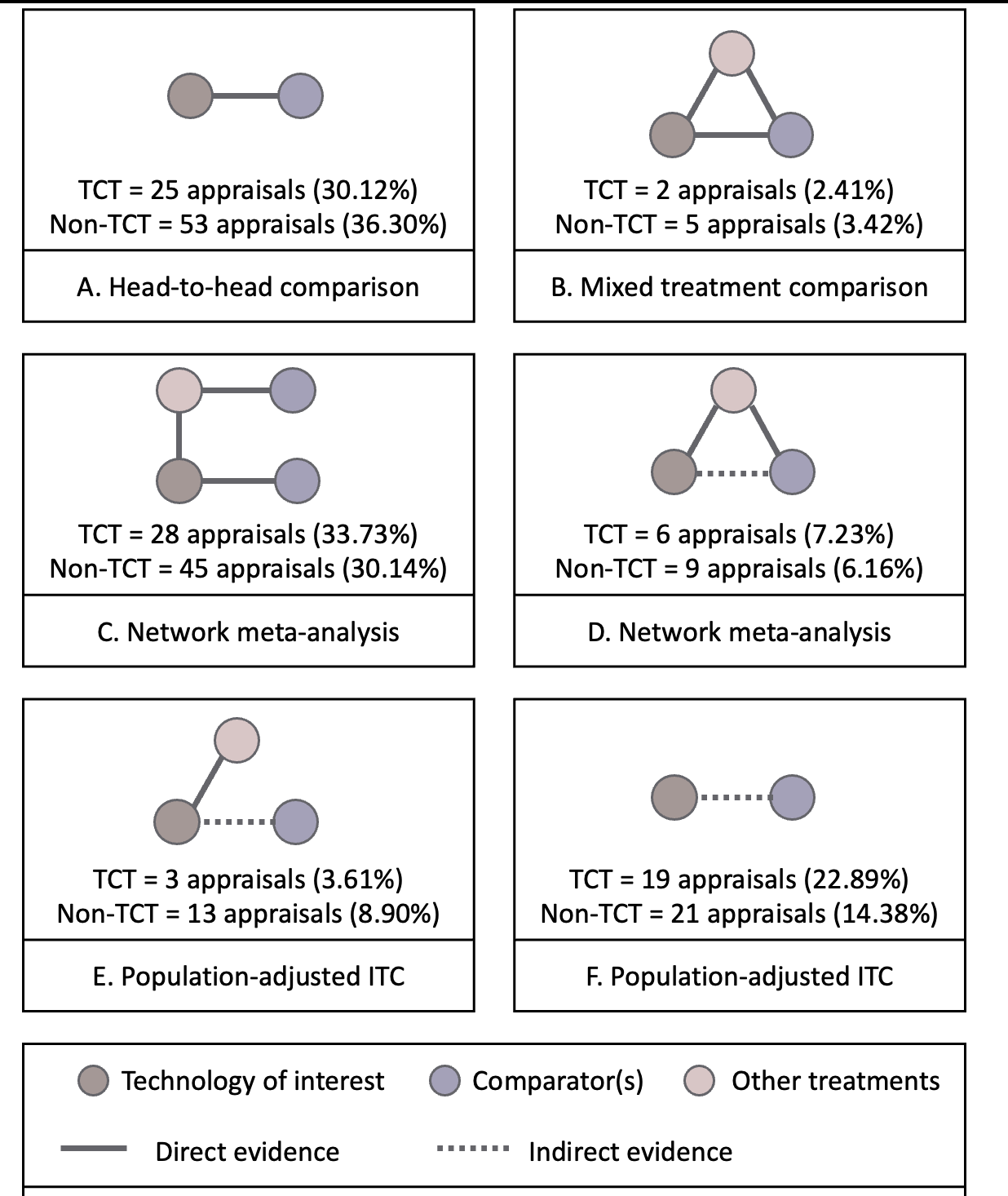 | Only available for some comparators | Yes, used | Yes, available | Yes | No (Naïve) |
|  |  |  |  |  |  | MAIC |
|  |  |  |  |  |  | STC |
|  |  |  |  |  |  | Other methods |
| MAIC: Matching adjusted indirect treatment comparisons, STC: Simulated treatment comparisons  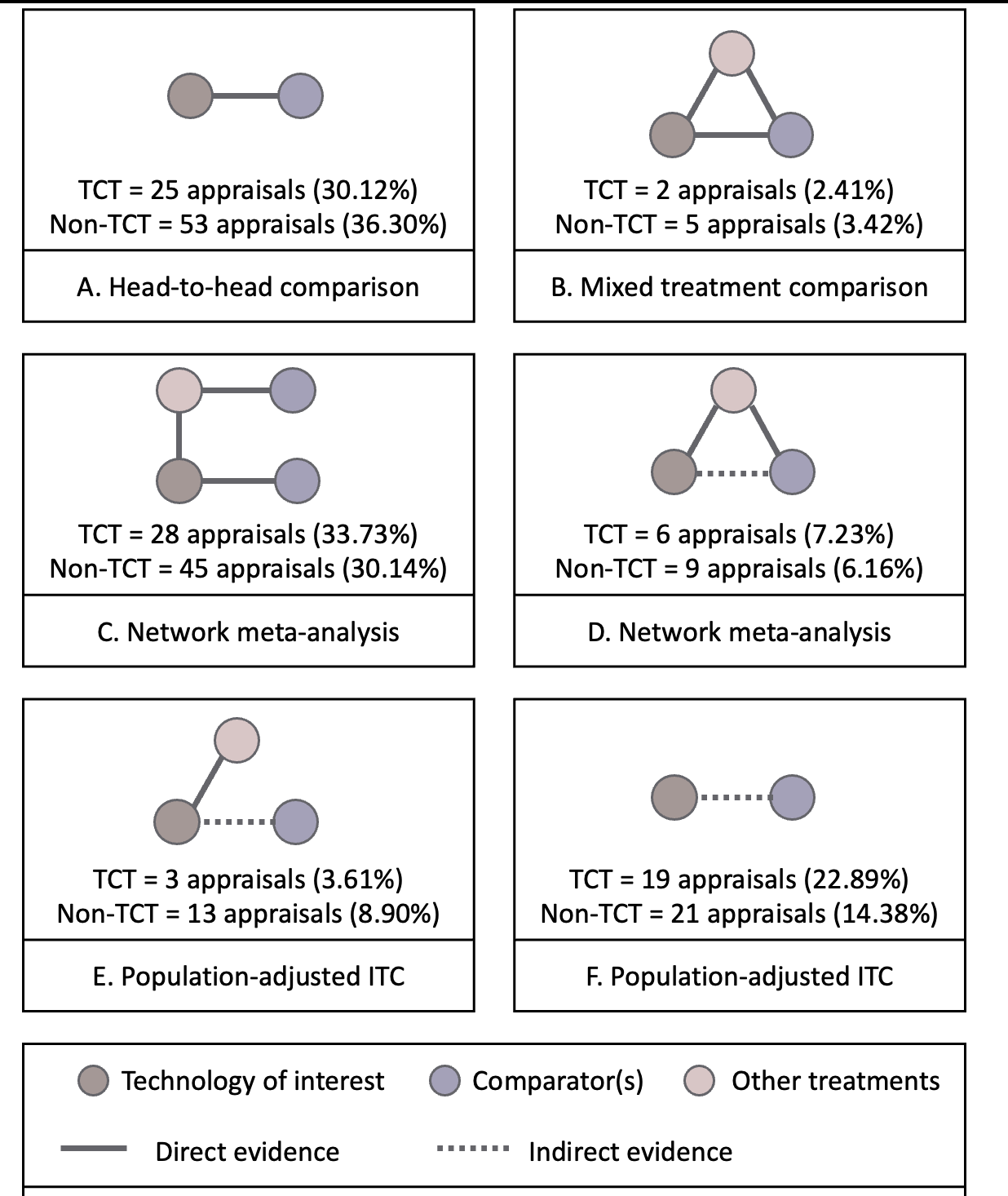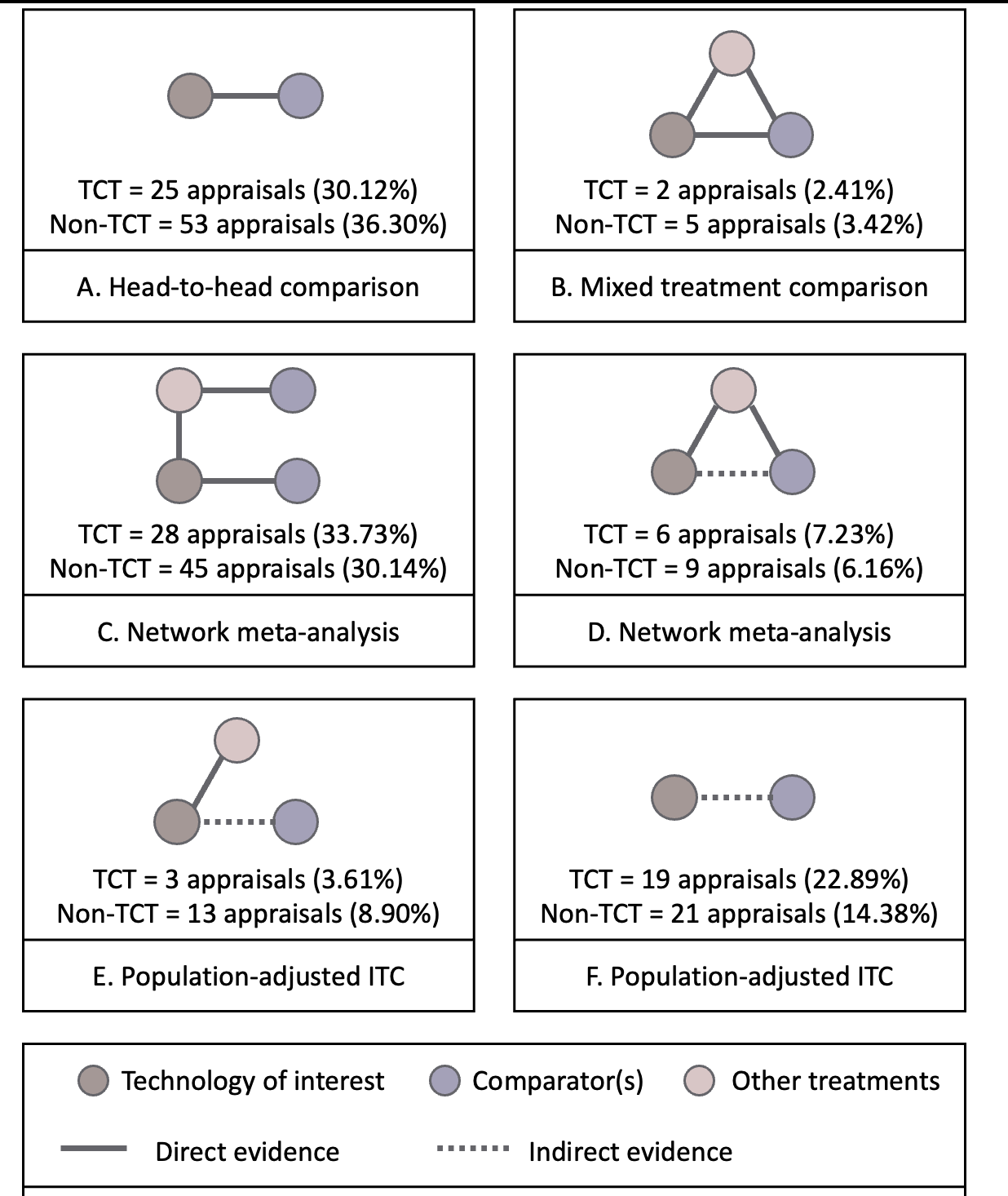 | | | | | | |

Appendix 2 Components in use of RWD in data extraction

| **Elements** |
| --- |
| Characteristics of population |
| Treatment sequence |
| Choice of comparators |
| Health state |
| Model cycle |
| Survival distribution (intervention) |
| Survival distribution (comparators) |
| Time-to-discontinuation (intervention) |
| Time-to-discontinuation (comparators) |
| Overall survival (OS) of intervention |
| Progression-free survival (PFS) of intervention |
| Response rate (intervention) |
| Time-to-progress (intervention) |
| Adverse event (intervention) |
| Overall survival (OS) of comparators |
| Progression-free survival (PFS) of comparators |
| Response rate (Comparators) |
| Time-to-progress (Comparators) |
| Adverse event (Comparators) |
| Transition probability |
| Health utility (generic measure) |
| Health utility (cancer specific measure) |
| Disutility |
| Resource use of health state cost |
| End-of-life resource use |
| Resource use of adverse event cost (intervention) |
| Volume of treatment (intervention) |
| Dose adjustment (intervention) |
| Resource use of adverse event cost (comparators) |
| Volume of treatment (comparators) |
| Dose adjustment (Comparators) |

Appendix 3 Pattern of use of RWD (Any use without considering non-parametric/parametric use)

| **Patterns** | **All appraisals** | **Non-targeted** | **Targeted** |
| --- | --- | --- | --- |
| No use of RWD | 37  (16.16%) | 25  (17.12%) | 12  (14.46%) |
| Estimating OS of intervention and comparators | 13  (5.68%) | 12  (8.22%) | 1  (1.20%) |
| Estimating end-of-life resource use | 12  (5.24%) | 4  (2.74%) | 8  (9.64%) |
| Estimating end-of-life resource use & resource use of health state costs | 8  (3.49%) | 4  (2.74%) | 4  (4.82%) |
| Estimating resource use of health state costs | 7  (3.06%) | 5  (3.42%) | 2  (2.41%) |
| Estimating OS of intervention and comparators and end-of-life resource use & resource use of health state costs | 6  (2.62%) | 4  (2.74%) | 2  (2.41%) |
| Estimating OS and PFS of intervention and comparators and resource use of health state costs | 5  (2.18%) | 3  (2.05%) | 2  (2.41%) |
| Validating survival distribution of intervention and comparators and estimating end-of-life resource use | 5  (2.40%) | 1  (0.68%) | 4  (4.82%) |
| Estimating OS and PFS of intervention and comparators | 5  (2.40%) | 1  (0.68%) | 4  (4.82%) |
| Estimating end-of-life resource use and dose adjustment of intervention and comparators | 4  (1.75%) | 4  (2.74%) | 0  (0%) |
| Estimating volume of treatment for intervention and comparators | 3  (1.31%) | 2  (1.37%) | 1  (1.20%) |
| Estimating OS of intervention and comparators and resource use of health state costs | 3  (1.31%) | 3  (2.05%) | 0  (0%) |
| Validating survival distribution of intervention and comparators | 3  (1.31%) | 1  (0.68%) | 2  (2.41%) |
| Choosing comparators | 3  (1.31%) | 3  (2.05%) | 0  (0%) |
| Choosing comparators and estimating resource use of health state costs | 3  (1.31%) | 2  (1.37%) | 1  (1.20%) |
| Others^*^ | 115  (48.91%) | 72  (49.33%) | 43  (48.21%) |
| Total | 229  (100%) | 146  (100%) | 83  (100%) |

^*^ The patterns are not listed because each represent a particular pattern of use of RWD only observed once or twice.

OS: Overall survival, PFS: Progression-free survival

Appendix 4 Pattern of use of RWD (Non-parametric use)

| **Pattern** | **All appraisals**  **n (%)** | **Non-targeted**  **n (%)** | **Targeted**  **n (%)** |
| --- | --- | --- | --- |
| No use of RWD | 136  (59.39%) | 91  (62.33%) | 45  (54.22%) |
| Validating survival distribution of intervention and comparators | 20  (8.73%) | 9  (6.16%) | 11  (13.25%) |
| Choice of comparators | 14  (6.11%) | 9  (6.16%) | 5  (6.02%) |
| Validating survival distribution of comparators | 13  (5.68%) | 8  (5.48%) | 5  (6.02%) |
| Treatment sequence | 7  (3.06%) | 3  (2.05%) | 4  (4.82%) |
| Characteristics of population | 7  (3.06%) | 5  (3.42%) | 2  (2.41%) |
| Validating survival distribution of intervention | 4  (1.75%) | 3  (2.05%) | 1  (1.20%) |
| Treatment sequence & validating survival distribution of intervention and comparators | 4  (1.75%) | 3  (2.05%) | 1  (1.20%) |
| Choice of comparator & validating survival distribution of intervention and comparators | 3  (1.31%) | 2  (1.37%) | 1  (1.20%) |
| Choice of comparator & validating survival distribution of comparators & time-to-discontinuation of comparators | 2  (0.87%) | 2  (1.37%) | . |
| Treatment sequence & time-to-discontinuation of intervention and comparators | 2  (0.87%) | 2  (1.37%) | . |
| Treatment sequence & validating survival distribution of comparators | 2  (0.87%) | . | 2  (2.41%) |
| Other^*^ | 15  (6.55%) | 9  (6.16%) | 6  (7.25%) |
| Total | 229  (100%) | 146  (100%) | 83  (100%) |

^*^ The patterns are not listed because each represent a particular pattern of use of RWD only observed once.

Appendix 5 Pattern of use of RWD (Parametric use)

| **Patterns** | **All appraisals** | **Non-targeted** | **Targeted** |
| --- | --- | --- | --- |
| No use of RWD | 55  (24.02%) | 37  (25.34%) | 18  (21.69%) |
| Estimating end-of-life resource use | 23  (10.04%) | 7  (4.79%) | 16  (19.28%) |
| Estimating OS of intervention and comparators | 17  (7.42%) | 15  (10.27%) | 2  (2.41%) |
| Estimating end-of-life resource use & resource use of health state costs | 14  (6.11%) | 7  (4.79%) | 7  (8.43%) |
| Estimating resource use of health state costs | 13  (5.68%) | 8  (5.48%) | 5  (6.02%) |
| Estimating OS & PFS of intervention and comparators | 10  (4.37%) | 4  (2.74%) | 6  (7.23%) |
| Estimating end-of-life resource use & dose adjustment of intervention and comparators | 9  (3.93%) | 7  (4.79%) | 2  (2.41%) |
| Estimating OS of intervention and comparators, end-of-life resource use & resource use of health state costs | 6  (2.62%) | 4  (2.74%) | 2  (2.41%) |
| Estimating OS & PFS of intervention and comparators & resource use of health state costs | 6  (2.62%) | 4  (2.74%) | 2  (2.41%) |
| Estimating volume of treatment for intervention and comparators | 4  (1.75%) | 3  (2.05%) | 1  (1.20%) |
| Estimating OS & PFS of comparators | 3  (1.31%) | 2  (1.37%) | 1  (1.20%) |
| Estimating OS of intervention and comparators & end-of-life resource use | 3  (1.31%) | 2  (1.37%) | 1  (1.20%) |
| Estimating OS of intervention and comparators & resource use of health state costs | 3  (1.31%) | 3  (2.05%) | . |
| Estimating OS & PFS of intervention and comparators, end-of-life resource use & resource use of health state costs | 3  (1.31%) | 2  (1.37%) | 1  (1.20%) |
| Other^*^ | 60  (26.20%) | 41  (28.08%) | 19  (22.89%) |
| Total | 229  (100%) | 146  (100%) | 83  (100%) |

^*^ The patterns are not listed because each represent a particular pattern of use of RWD only observed once or twice.

OS: Overall survival, PFS: Progression-free survival
